# PROVIT: Supplementary Probiotic Treatment and Vitamin B7 in Depression—A Randomized Controlled Trial

**DOI:** 10.3390/nu12113422

**Published:** 2020-11-08

**Authors:** Eva Z. Reininghaus, Martina Platzer, Alexandra Kohlhammer-Dohr, Carlo Hamm, Sabrina Mörkl, Susanne A. Bengesser, Frederike T. Fellendorf, Theressa Lahousen-Luxenberger, Birgitta Leitner-Afschar, Helmut Schöggl, Daniela Amberger-Otti, Walter Wurm, Robert Queissner, Armin Birner, Valerie S. Falzberger, Annamaria Painold, Werner Fitz, Martina Brunnmayr, Alexandra Rieger, Jolana Wagner-Skacel, Alexander Maget, Renate Unterweger, Karin Schwalsberger, Bernd Reininghaus, Melanie Lenger, Thomaz F. S. Bastiaanssen, Nina Dalkner

**Affiliations:** 1 Department of Psychiatry and Psychotherapeutic Medicine, Medical University of Graz, 8036 Graz, Austria; eva.reininghaus@medunigraz.at (E.Z.R.); martina.platzer@medunigraz.at (M.P.); alexandra.kohlhammer-dohr@medunigraz.at (A.K.-D.); carlo.hamm@medunigraz.at (C.H.); susanne.bengesser@medunigraz.at (S.A.B.); frederike.fellendorf@medunigraz.at (F.T.F.); theresa.lahousen@medunigraz.at (T.L.-L.); birgitta.leitner-afschar@klinikum-graz.at (B.L.-A.); h.schoeggl@medunigraz.at (H.S.); daniela.amberger-otti@klinikum-graz.at (D.A.-O.); walter.wurm@medunigraz.at (W.W.); robert.queissner@medunigraz.at (R.Q.); armin.birner@medunigraz.at (A.B.); valerie.falzberger@gmail.com (V.S.F.); annamaria.painold@medunigraz.at (A.P.); werner.fitz@medunigraz.at (W.F.); alexandra.rieger@medunigraz.at (A.R.); alexander_maget@chello.at (A.M.); renate.unterweger@medunigraz.at (R.U.); karin.schwalsberger@stud.medunigraz.at (K.S.); melanie.lenger@medunigraz.at (M.L.); nina.dalkner@medunigraz.at (N.D.); 2 TZ-Justus Park, 4540 Bad Hall, Austria; Martina.brunnmayr@bva.at (M.B.); berndreininghaus@web.de (B.R.); 3 Department of Medical Psychology and Psychosomatics, Medical University of Graz, 8036 Graz, Austria; jolana.wagner-skacel@medunigraz.at; 4 Department of Anatomy and Neuroscience, University College Cork, T12 YN60 Cork, Ireland; thomaz.bastiaanssen@ucc.ie; 5 APC Microbiome Ireland, University College Cork, T12 YN60 Cork, Ireland

**Keywords:** depression, affective disorders, gut-brain-axis, probiotics, inflammation, microbiome, biotin

## Abstract

Gut microbiota are suspected to affect brain functions and behavior as well as lowering inflammation status. Therefore, an effect on depression has already been suggested by recent research. The aim of this randomized double-blind controlled trial was to evaluate the effect of probiotic treatment in depressed individuals. Within inpatient care, 82 currently depressed individuals were randomly assigned to either receive a multistrain probiotic plus biotin treatment or biotin plus placebo for 28 days. Clinical symptoms as well as gut microbiome were analyzed at the begin of the study, after one and after four weeks. After 16S rRNA analysis, microbiome samples were bioinformatically explored using QIIME, SPSS, R and Piphillin. Both groups improved significantly regarding psychiatric symptoms. *Ruminococcus gauvreauii* and *Coprococcus 3* were more abundant and β-diversity was higher in the probiotics group after 28 days. KEGG-analysis showed elevated inflammation-regulatory and metabolic pathways in the intervention group. The elevated abundance of potentially beneficial bacteria after probiotic treatment allows speculations on the functionality of probiotic treatment in depressed individuals. Furthermore, the finding of upregulated vitamin B6 and B7 synthesis underlines the connection between the quality of diet, gut microbiota and mental health through the regulation of metabolic functions, anti-inflammatory and anti-apoptotic properties. Concluding, four-week probiotic plus biotin supplementation, in inpatient individuals with a major depressive disorder diagnosis, showed an overall beneficial effect of clinical treatment. However, probiotic intervention compared to placebo only differed in microbial diversity profile, not in clinical outcome measures.

## 1. Introduction

Depression is one of the most common mental disorders and a leading cause of global socioeconomic burden of disease worldwide, as it may result in significant disabilities in affected patients. Inflammation is a major pathophysiological pathway associated with affective disorders [1]. In this context, the microbiota-gut-brain-axis (MGBA) has gained increasing interest as a bidirectional communication system between the gut and brain, especially as microbiota metabolites mediate the inflammatory pathways. Microbiota and their compounds release pro-inflammatory cytokines, change intestinal permeability and alter immune response; changes that may lead to disturbances in mental health [2]. According to recent research, microbiota are likely to have effects on brain function and behavior, including affect, motivation and higher cognitive functions [3,4,5,6]. Furthermore, animal-based research found specific intestinal microbes to be beneficial in brain development and microglia [7,8]. A disbalance of the communication between gut microbiota and the central nervous system (CNS) may, thus, contribute to neuropsychiatric disorders such as depression [4,9]. Nevertheless, not all recent literature seems to agree univocally on this point. In systematic reviews, studies investigating connections between microbiome and brain as well as emotional health, conclude that those remain largely unexplored [10].

The microbes living in human gut form an ecosystem. Attributes to define this system are abundance, describing the plenitude of a taxon, and diversity, which means the heterogeneity of taxa within the system. Alpha diversity refers to the number of different species within one given sample, whereas beta diversity is defined by differences in the composition of two given samples. Besides microbe composition, the intestinal mucosal barrier is also of major importance in the interaction between gut and brain. This barrier is formed by epithelial cells and a mucus layer, which are conjunct via tight junctions that prevent and control the paracellular passing of agents [11]. Zonulin is an endogenous protein with the ability to open tight junctions and to increase intestinal permeability [12] consecutively.

One strategy to modulate the gut microbiome and, therefore, potentially change the communication between the gut and brain is the intake of probiotic supplements. Probiotics are defined as living micro-organisms that reconstitute the gastrointestinal barrier [13,14]. If taken in certain amounts, beneficial health status and decrease of potentially pathogenic gut bacteria, as well as a positive influence on the immune system, have been found as summarized in different reviews and meta-analyses [6,13,15,16]. Animal experiments demonstrated that probiotics also influenced mice’s behavior and alleviated symptoms of depression or anxiety [9,15].

Nevertheless, studies on probiotics in individuals with psychiatric disorders are currently rare. In a recent meta-analysis, 19 double-blind, randomized, placebo-controlled trials on the effect of probiotics on depressive symptoms were published between 2010 and 2019 [17,18]. Notably, only three of them included individuals with major depressive disorder (MDD) [19,20,21], while the other studies were conducted with healthy controls or other clinical populations (e.g., irritable, bowel syndrome, diabetes with coronary heart disease, fibromyalgia) [6,13,15,17,18]. Akkasheh et al. [19] included 40 patients with MDD (age 20–55 years) receiving an eight-week intervention with *Lactobacillus (L.) acidophilus*, *L. casei* and *Bifidobacterium (B.) bifidum* versus placebo. Consumption of probiotic supplement improved Beck Depression Inventory (BDI) scores in this study. Ghorbhani et al. [20] investigated the efficacy of six weeks synbiotic supplementation in 40 moderate depressive individuals treated with fluoxetine. Synbiotic supplementation included *L. acidophilus*, *L. casaei*, *L. bulgarigus*, *L. rhamnosus*, *B. breve*, *B. longum* and *S. thermophilus*, and 100 mg fructooligosaccharide as prebiotic. At the end of the study, the synbiotic group had a significantly decreased Hamilton depression scale (HAMD) score compared to the placebo. In the study of Kazemi et al. [21], the effect of probiotic supplementation (*L. helveticus* and *B. longum*) was compared to prebiotics and placebo in 81 individuals with depression over a time period of eight weeks. Individuals in the probiotic supplementation group had a significant decrease in BDI scores compared to the other groups.

Improvements in depressive symptoms were significantly higher in individuals with MDD treated with probiotics than in those receiving placebo in patients (for a review see [18]). In another review [22], one more study has been identified including an intervention with probiotics compared to placebo in 60 individuals with depression [23]. Furthermore, in a pilot study, our study group could show a significantly reduced cognitive reactivity to sad mood in 27 individuals with euthymic bipolar disorder receiving probiotic treatment over a period of three months, indicating that participants under probiotic supplementation perceived themselves to be less distracted by ruminative thoughts [24].

Novel, innovative and personalized treatment options in individuals with affective disorders are urgently needed. Preclinical experiments as well as clinical studies point to the importance of the MGBA, and more specifically to the intestinal microbes in the pathogenesis of depressive disorders. Modulation of these microbes might have an impact on the development and continuation of depressive symptoms.

We, therefore, conducted a double-blind, placebo-controlled, randomized study in individuals with depressive symptoms receiving either the multistrain probiotic “Omnibiotic Stress Repair^®^” plus biotin or a placebo plus biotin over 28 days in addition to standard antidepressive treatment. The study aimed to analyze and compare the effects on clinical parameters as well as on intestinal microbiota between the two groups.

We hypothesized that depressed individuals treated with the probiotic supplement in addition to standard inpatient treatment would:

1. Experience significantly higher improvement in psychiatric symptoms than individuals treated with placebo after one month;
2. Show significant changes in intestinal barrier function (measured by zonulin) in comparison to treatment with placebo after one month;
3. Have significant changes in microbiome analysis (alpha and beta diversity, global differential abundance, Piphillin-Analysis) in comparison to individuals with placebo treatment after one week as well as after one month.

## 2. Materials and Methods

The PROVIT study was performed as a monocentric, randomized, placebo-controlled study, approved by the local ethics board (EK 29-235 ex 16/17) and registered at clinicaltrials.com (NCT03300440). The term PROVIT was created due to the intake of probiotics and vitamin B7. All subjects recruited were inpatients of the Department of Psychiatry and Psychotherapeutic Medicine of the Medical University Graz and provided written informed consent after previous written and verbal information.

Study visits were performed at the time of inclusion (t0), after one week (t1; only stool) and after 28 days (t2; ±3 days). Clinical visits included collection of fasting blood, stool, psychological and cognitive testing as well as a clinical interview including side effects of (study) medication and clinical symptoms. For results of the gene expression data of the study, see [25].

**Figure 1:**
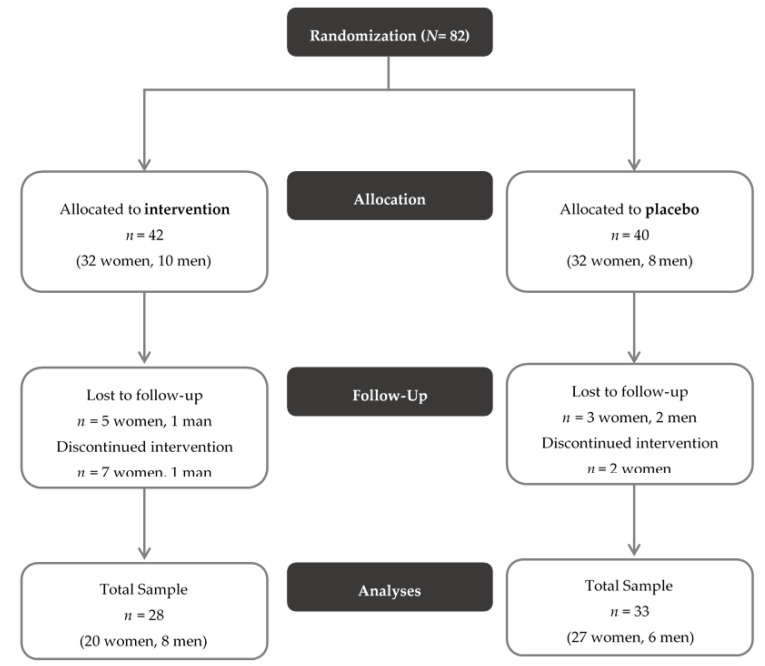
CONSORT Flow Diagram of the PROVIT study. Exclusion criteria consisted of acute suicidality, lack of consent, pregnancy or breastfeeding, severe active drug dependence (i.e., alcohol, benzodiazepines, morphine), other currently active severe cerebral organic diseases (e.g., epilepsy, brain tumor), severe skull- brain trauma/brain surgery in the past, known florid tumor disease, congenital/infantile mental disability, moderate/severe dementia, severe florid autoimmune diseases or current immunosuppression (e.g., lupus erythematosus, HIV, multiple sclerosis), antibiotic therapy within the last month, chronic laxative abuse, acute infectious diarrheal disease, regular intake of butyrate-containing or probiotic supplements in the last year, intake of (other) probiotics and prebiotics or butyrate preparations during the entire trial or within the last month and intake of antibiotics or prebiotics during the entire trial or within the last month.

The study design of the PROVIT study is depicted in Figure 1.

Psychiatric diagnosis was evaluated by a psychiatrist using the Mini International Neuropsychiatric Interview M.I.N.I. (Mini-International Neuropsychiatric Interview) [26]. Inclusion criteria demanded a current diagnosis of a depressive episode and age between 18 and 75 years.

During the intervention phase, patients did not take other prebiotics, antibiotics or laxatives. In accordance with the treatment program of the Department of Psychiatry and Psychotherapeutic Medicine, participants received treatment as usual, which included physiotherapy, occupational therapy, psychopharmacological therapy and psychotherapy. If required, pharmaceuticals were changed or adapted.

### 2.1. Study Medication

The probiotic supplement as well as the placebo was provided by the “Institute Allergosan,” producer of the product was Winclove BV, Amsterdam, Netherlands. OMNi-BiOTiC^®^ Stress Repair is a commercial dietary supplement and includes nine bacterial strains with at least 7.5 billion organisms per 1 portion (3 g). Bacterial strains in “OMNi-BiOTiC^®^ Stress Repair” are *B. bifidum* W23, *B. lactis* W51, *B. lactis* W52, *L. acidophilus* W22, *L. casei* W56, *L. paracasei* W20, *L. plantarum* W62, *L. salivarius* W24 and *L. lactis* W19. In addition, 125 mg D-Biotin (vitamin B7), 30 mg of common horsetail, 30 mg of fish collagen and 30 mg of keratin plus matrix was added to the probiotic product. Both groups received biotin additionally (but included in the study medication), due to ethical considerations.

Both groups of patients should receive a substance that might be beneficial for them. Therefore, the placebo product included 125 mg D-Biotin (vitamin B7), 30 mg of common horsetail, 30 mg of fish collagen and 30 mg of keratin plus matrix. The placebo product had the same color, consistency and taste as the probiotic product.

Matrix included maize starch, maltodextrin, inulin, potassium chloride, magnesium sulfate, fructooligosaccharides (FOS), enzymes (amylases) and manganese sulfate.

Patients received the probiotic supplement “OMNi-BiOTiC^®^ Stress Repair”, Allergosan, Graz, Austria or the placebo product (stirred with water and with obeying an activation time of ten minutes) from a member of the study team daily at 7 AM before breakfast over a period of 28 days. Participants as well as the whole study team were blinded to the study condition assignment. Randomization was done with www.randomization.com and 96 subjects were randomized into 24 blocks of 4 to assign the individuals to either intervention or placebo group.

The manufacturer assembled the test product packages in accordance with the list provided by the person responsible for randomization with the group assignments (one package per study participant number). Each package was identified with the relevant study participant number (1, 2, 3, etc.) and could only be distinguished from all other packages by this number. The person responsible for randomization and all unblinded employees of the manufacturer were bound to secrecy towards third parties. The group assignment of the study participants took place in the ratio group probiotic: group placebo = 1:1. The whole study team was blinded to the randomization until the end of the study.

### 2.2. Demographics and Scales of PROVIT

The PROVIT study included assessment of psychiatric symptoms using the HAMD [27], the BDI-II [28] and the Symptom Checklist-90-Revised (SCL-90) [29]. The HAMD is an external assessment performed by psychiatrists to evaluate the severity of depressive symptoms, including 21 items on a three-point or five-point Likert-type scale. The BDI-II is a self-report inventory to assess the severity of depressive symptoms in the last two weeks with 21 items, each answer being scored on a scale value of 0 to 3 points. The SCL-90-R is a 90-item self-report inventory to assess psychological symptoms and psychological distress. Cognitive, physical and emotional symptoms of distress and overall distress in the last seven days were rated on a five-point Likert scale with nine scales (Somatization, Obsessive-compulsive disorder, Interpersonal sensitivity, Depression, Anxiety, Hostility, Phobic anxiety, Paranoid ideation and Psychoticism) and three global parameters: Global Severity Index (GSI), which measures fundamental psychological distress, Positive Symptom Distress Index (PSDI), which captures response intensity, and Positive Symptom Total (PST), which indicates the number of symptoms. The Mania Self Rating Scale (MSS; [30]) was used to measure subjective manic symptoms. “The gastrointestinal quality of life questionnaire” (GLQI; [31]) is a self-rating questionnaire which was used to measure the quality of life concerning gastrointestinal symptoms.

Clinical and demographic parameters including age, weight, height, body mass index (BMI), sex and medication were also documented. Psychopharmacological substance categories included anticonvulsants, atypical antipsychotics, benzodiazepines and hypnotics, glutamatergic antidepressants, low potency antidepressants, melatonin-like antidepressants, mixed preparation of antidepressant and antipsychotic, noradrenergic and specific serotonergic antidepressants, norepinephrine-dopamine reuptake inhibitors (NDRIs), selective serotonin reuptake inhibitors (SSRIs), serotonin-norepinephrine reuptake inhibitors (SNRIs) as well as tri- and tetracyclic antidepressants. Microbiome analysis (16S rRNA-sequencing) from stool was performed at time points t0, t1 and t2, while fasting blood analysis and cognitive testing was performed at t0 and t2.

Questionnaires that have not yet been analyzed in the current analysis, but were still included, were the “Bristol Stool Scale” [32], cognitive diagnostic and the “Food Craving Inventory” [33].

### 2.3. Statistics of Clinical Data

Sample size was determined as calculated by random number calculation. Descriptive data were assessed by using means (M) and standard deviations (SD) as well as percentages of the respective variable. To test if there were differences between t0 and t2 in clinical variables during the course of probiotic/placebo intake, analyses of variance with repeated measures (RM-ANOVAS) were conducted. All analyses were performed using IBM SPSS 22. Error probabilities below *p* < 0.05 were accepted to denote statistical significance.

### 2.4. Zonulin Analysis

Zonulin concentration was measured with the IDK^®^ Zonulin ELISA assay from Immundiagnostik AG (Bensheim, Germany) in serum samples that were stored at −80 °C prior to analysis. Briefly, samples were incubated with a biotinylated Zonulin family peptide (ZFP) tracer. In the second incubation step, peroxidase-labelled streptavidin binds to the biotinylated ZFP tracer. Following a washing step to remove unbound components, the peroxidase substrate tetramethylbenzidine was added. Then, the enzyme reaction was stopped by the addition of acid. The resulting chromogenic compound was measured photometrically at 450 nm. The intensity of the color was inversely proportional to the concentration of the measured analytes.

### 2.5. Microbiome Analysis

One gram of the collected stool sample was immediately stored in a −80 °C-freezer. Sequence analysis was done according to the supplier’s recommendations. The workflow for microbiome analysis with Illumina MiSeq has already been described in detail in Klymiuk et al., 2017 [34]. The Magna Pure LC DNA III Isolation Kit (Bacteria, Fungi) (Roche, Mannheim, Germany) was used to extract DNA according to manufacturer’s instructions. The hypervariable V3–V4 regions of the bacterial 16S rRNA gene were amplified with Polymerase-chain-reaction (PCR) from fecal total DNA using the target specific primers MyOv3v4F—CCTACGGGNGGCWGCAG and MyOv3v4R—GACTACHVGGGTATCTAATCC [35]. Two µL of the total DNA were used in a 25 µL PCR reaction in triplicates with the FastStart High Fidelity PCR system (Sigma, Germany). Cycling condition were of initial denaturation at 95 °C for 3 min, followed by 30 cycles of 95 °C for 45 s, 55 °C for 45 s, 72 °C for one minute and a final elongation step at 72 °C for seven minutes. The resulting amplification products were visualized on a 1.5% agarose gel and pooled, indexed and purified as described in Klymiuk et al., 2017 [34]. The final library was sequenced at ZMF Core Facility Molecular Biology in Graz, Austria, using an Illumina MiSeq desktop sequencer with v3 chemistry and 600 cycles (2 × 300). FASTQ files were used for data analysis. The data for this study have been deposited in the European Nucleotide Archive (ENA) at EMBL-EBI under accession number PRJEB40986 (http://www.ebi.ac.uk/ena/data/view/PRJEB40986).

### 2.6. Bioinformatics

Unmapped bam files were used as input for bioinformatics. To analyze the microbial community structure and taxonomic diversity, the obtained raw reads were processed using Quantitative Insights Into Microbial Ecology (QIIME, v1.9.1) scripts on the galaxy server of the Medical University of Graz (galaxy.medunigraz.at). The produced sequences were quality checked with FASTQC, denoised and dereplicated using standard parameters (dada2 denoise-paired) and then aligned against the SILVA 132 release database. Good-quality sequences were pre-clustered and chimeric sequences were removed. The taxonomic assignment was carried out with the RDP Classifier [36] using Naive Bayes classification with default parameters. De novo multiple sequence alignment was done with MAFFT and a phylogenetic tree was constructed with FastTree. A biom table was constructed for downstream analyses.

### 2.7. Statistical Analysis and Visualization of Microbiome Data

Microbiome analysis was performed in R (v3.6) and Rstudio (1.2.1555) (R-foundation, Vienna, Austria). Data visualization was performed using the ggplot2 library. Unless stated otherwise, descriptive results of continuous variables are expressed as M and SD for Gaussian distributed variables. To measure and compare levels of alpha-diversity between intervention and placebo group, Chao1-diversity Index, Simpson’s Index and Shannon’s Index were calculated with the iNEXT library [37]. Microbiome data are compositional, and as such, CoDa was adhered to [38]. Differential abundance analysis was done using the ALDEx2 library [39] in R, and beta diversity, or between-sample diversity, was calculated using Aitchison distance, also using ALDEx2. The adonis() function in the vegan library was used to assess differences in beta-diversity [40]. Levels of statistical significance were set to *p* < 0.05. Principal Component Analysis (PCA) was used to visualize beta diversity. To correct for multiple testing in tests regarding specific microbiota, KEGG orthologues or pathways, Storey’s *q*-value post-hoc procedure was performed with a q-value of 0.1 as a cut-off [41].

Piphillin was used for functional analysis to predict the metagenomic content of samples [42]. To ensure power and reliability of the downstream analysis, data were filtered based on a minimum number of observed genera (7500) and all three time points that were available per participant.

## 3. Results

Out of 82 patients included, 82 participants provided written informed consent for the study. Of them, 61 individuals were included in the study (see Figure 1) and completed at least the baseline study visit (t0, *n* = 28 probiotics, *n* = 33 placebo). There were no significant differences in the clinical parameters between the two groups, with the exception that there were significantly more smokers in the placebo group than in the probiotics group. The sample’s baseline values are depicted in Table 1. In addition, stool samples for all three time points (t0, t1, t2) were available from 53 participants, who, therefore, were included in microbiome analysis (*n* = 26 probiotics, *n* = 27 placebo). Concerning medication, there was no significant difference in the number of substance classes (χ2(2) = 3.169, *p* = 0.205) between the two groups. In the probiotic group six individuals were not pre-medicated, whereas in the placebo group, only two individuals were not pre-medicated. Fourteen and nineteen participants, respectively, took medication from one or two different substance categories and eight and twelve individuals, respectively, took drugs from three or more distinct substances.

### 3.1. Clinical Parameters

Both groups improved significantly over time in psychiatric symptoms. There was no significant difference in the changes between the probiotics and placebo group in any of the psychiatric scales (see Table 2).

### 3.2. Anthropometric Data

BMI and waist to hip ratio were stable over time and there were no significant group*time interactions.

### 3.3. Zonulin

Zonulin did not significantly change over time, nor there were any group * time effects (see Table 3).

### 3.4. Microbiome Analysis

#### 3.4.1. Alpha-Diversity

Before treatment with study medication (t0), participants of the probiotics group did not show any significant difference compared to the placebo group in alpha diversity indices (number of observed species (F(1,51) = 0.004, *p* = 0.948), Chao-1-diversity index (F(1,51) = 0.004, *p* = 0.951), Simpson index (F(1,51) = 0.001, *p* = 0.995) and Shannon index (F(1,51) = 0.003, *p* = 0.959).

One week after the start of the probiotic intervention (t1), no significant differences in measures of alpha-diversity, such as number of observed species (F(1,51) = 0.011, *p* = 0.919), Chao-1-diversity index (F(1,51) = 0.011, *p* = 0.917), Simpson index (F(1,51) = 0.528, *p* = 0.471) and Shannon index (F(1,51) = 0.113, *p* = 0.738), were detected between the probiotics and the placebo group.

**Figure 2:**
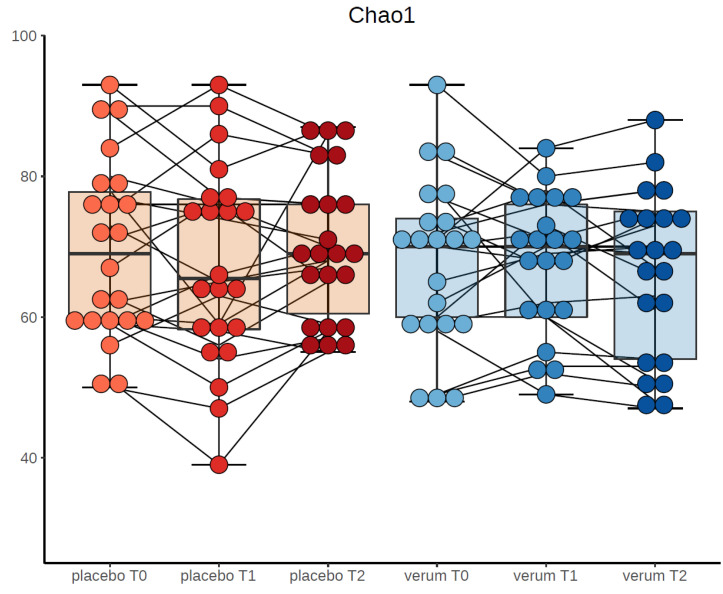
Chao-1-diversity at the three time points in probiotic and placebo group.

Furthermore, there were no significant differences of alpha-diversity indices at the end point of the study (t2) between the probiotics and placebo group (number of observed species (F(1,51) = 1.213, *p* = 0.276), Chao-1-diversity index (F(1,51) = 1.203, *p* = 0.278), Simpson index (F(1,51) = 0.247, *p* = 0.621) and Shannon index (F(1,51) = 0.310, *p* = 0.580). Figure 2 depicts number of observed species, Chao-1 diversity and Shannon index for verum and placebo group divided by time points.

#### 3.4.2. Beta-Diversity

On a beta-diversity level, we could not detect a significant difference at t0 between probiotics and placebo groups (R2 = 0.032, *p* = 0.071), while there was a significant difference at t1 (R2 = 0.038, *p* = 0.009) as well as t2 (R2 = 0.035, *p* = 0.026) between the groups.

We also found a significant difference between the probiotics and placebo group for the total samples of t1 and t2 (R2 = 0.02112, *p* = 0.001). Individuals in the probiotics group differed significantly with regard to beta diversity compared to the placebo group.

**Figure 3:**
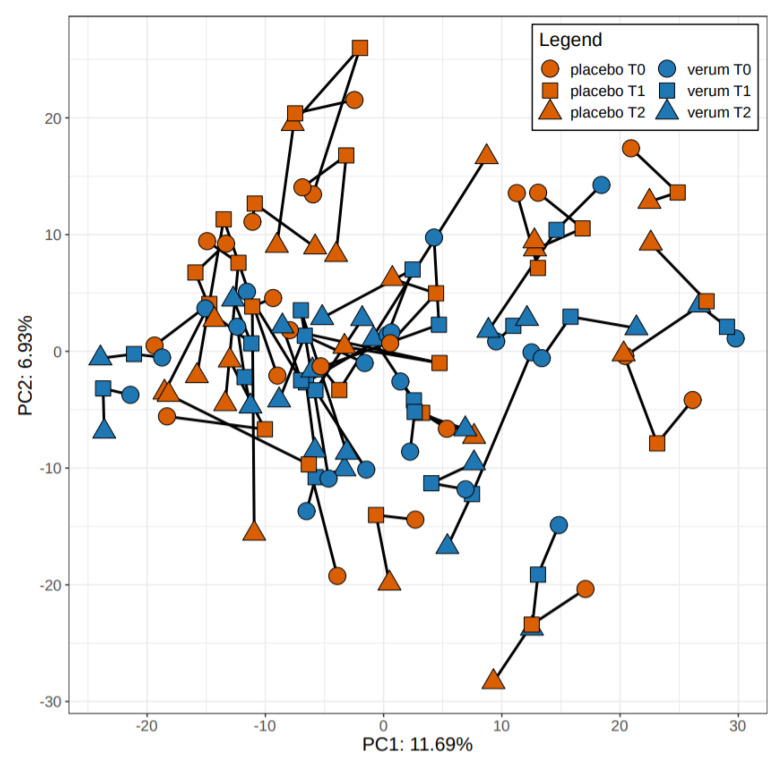
Principal component analysis of participants of verum and placebo group at all three time points.

Figure 3 depicts the principal component analysis of participants of probiotics and placebo group at all three time points.

#### 3.4.3. Global Differential Abundance

In terms of global differential abundance, we found a significant increase in the *Ruminococcus (R.) gauvreauii* group in the probiotics group at t1 (*q* = 0.098, effect size = 0.748) and at t2 (*q* = 0.092, effect size = 0.809). Additionally, we found an increase of the taxonomically related *Coprococcus 3* in the probiotics group after one month of treatment at t2 (*q* = 0.15058645, effect size = 0.4241559).

#### 3.4.4. Piphillin-Analysis

Functional inferences were calculated with Piphillin [36]. Table 4 gives an overview about relevant pathways showing a significant difference between probiotics and placebo at t2 (*q* < 0.005). All pathways were upregulated in the intervention group.

## 4. Discussion

The study aimed to perform a randomized placebo-controlled trial in individuals with current depression receiving the multistrain probiotic “Omnibiotic Stress Repair^®^” compared to a placebo product over a study period of 28 days in addition to treatment as usual. We investigated the effects on (1) psychiatric symptoms, (2) intestinal barrier function and (3) microbiome between the two groups.

Most importantly, significantly altered beta-diversity, as well as a significant increase in the *R. gauvreauii* group in terms of global differential abundance, was evident already after one as well as after four weeks in individuals receiving probiotic supplement in comparison to the placebo group. Furthermore, we found a significant increase in the taxonomically related *Coprococcus 3* in the probiotics group at the end of the study (28 days of intake) in comparison to placebo. However, we did not find significant differences concerning alpha diversity. The results showed a significant improvement in psychiatric symptoms in both groups over time but no interaction effects. Accordingly, Zonulin, as a marker of intestinal barrier function, showed no significant changes.

In recent studies, *Coprococcus bacteria* and *Faecalibacterium* were consistently associated with higher quality of life indicators. Moreover, in the Flemish gut flora project, one of the most extensive studies concerning microbiome research that investigated more than a thousand individuals with depression compared to healthy controls, *Coprococcus* species were found to be depleted in individuals with depression [43]. This is especially interesting in the context of our study results, as *Coprococcus* increased during probiotic intake in our study. Coprococcus is a family member of Lachnospiraceae and a butyrate producer [44]. In a study with prebiotics, it was also found that *Ruminococcaceae* increased due to fermentable fiber in diet together with a higher production of butyrate. Therefore, butyrate could be one functional aspect linking the higher abundance of *Coprococcus* and *R. gauvreauii* [45]. However, this remains speculative until more studies are done on this subject [46].

Further associations with our results were shown by a meta-analysis by Sanada et al. [22]. Sanada et al. found, in line with our study results, on the genus level *Coprococcus* and *Ruminococcus*, in addition to *Faecalibacterium*, *Bifidobacterium* and *Escherichia* to be decreased in individuals with depressive disorders receiving probiotics in addition to antidepressive therapy. Findings of the diversity assessments were inconsistent. Reduced abundance of *Ruminococcus* and *Faecalibaceterium* has further been found previously by our study group in individuals with bipolar depression compared to healthy controls [47]. As some changes between probiotic treatment and placebo were already seen after one week in this study, the influence on microbiota composition might already start early in the treatment period.

There is an increasing awareness of variations in probiotic strains and their impact on host outcomes in probiotic studies. Moreover, in existing studies, the strain of probiotic, the dose and duration of treatment varied widely. In a consensus statement of the International Scientific Association for Probiotics and Prebiotics [48], some general benefits were described for the well-studied species *B*. (*adolescentis*, *animalis*, *bifidum*, *breve* and *longum)* and *L.* (*acidophilus*, *casei*, *fermentum*, *gasseri*, *johnsonii*, *paracasei*, *plantarum*, *rhamnosus* and *salivarius*). The product used in the present study included multiple strains of *Bifidobacterium* and *Lactobacillus*, which have shown beneficial effects in respect to mental health already in previous studies [6]. Interestingly, we nevertheless did not find changes in *Lactobacillus* or *Bifidobacteria*, the two genera represented in the polybiotic supplement. Nevertheless, this does not necessarily invalidate the study by any means, as there are many prebiotic compounds in the supplement (like FOS). Furthermore, it is well known that the microbiome is a complex ecosystem; thus, it is likely that the introduction of *Lactobacillus* or *Bifidobacteria* would naturally result in the changes we observed without changing the actual abundance of those genera.

Our results of the missing significance with regards to alpha diversity underlines the findings of other probiotic studies showing that probiotic administration did not significantly alter gut microbiota alpha diversity despite probiotic proliferation [49,50].

Looking at the KEGGs pathways in our study (Table 4), high effect sizes were found in inflammatory as well as metabolic pathways. Furthermore, vitamin B6/B7 as well as B1 metabolism was upregulated in the intervention group. This is of special interest, as both groups of patients received vitamin B7 (biotin) in addition to the probiotic supplementation or placebo. This was due to ethical considerations, as both groups of patients should receive a substance that might be beneficial for them.

Vitamin B6 metabolism is known to be involved in the pathophysiology of psychiatric diseases and in its active form, pyridoxal 5′-phosphate (PLP), plays a role in the control of plasma homocysteine concentration [51]. There is evidence that homocysteine might be involved in the biological underpinnings of psychiatric disorders as it interacts with N-methyl-D-aspartate (NMDA) receptors, causes oxidative stress, induces apoptosis, triggers mitochondrial dysfunction and leads to vascular damage [52]. In this context, homocysteine is known to serve as an atherosclerotic factor. This is of special interest as it is known that individuals with psychiatric disorders in a high proportion suffer from somatic disorders compared to mentally healthy individuals. In this context it appears even more interesting that different “metabolic pathways” including glucose and fat metabolism were significantly different between the verum and control group. This is of note, as we know that a high percentage of individuals with depression suffer from somatic comorbidities leading to decreased quality of life as well as highly reduced life expectancy [53]. In future studies, a focus on somatic comorbidities in individuals with depressive symptoms receiving probiotic supplements might help to further understand this association better in the context of the MGBA as already suggested by a review in 2017 [54]. They concluded that a manipulation of the gut microbiota with probiotics may open a new avenue for the prevention and treatment of MDD and associated comorbidities and that these beneficial effects could attenuate metabolic endotoxemia.

Unexpectedly, but even more interesting, seems the result of the upregulation of “vitamin B7 (biotin) metabolism.” It is known that some gut microbiota can produce biotin themselves, while others cannot or even consume biotin and thereby decrease biotin-availability [55,56]. The balance between biotin-producing and -consuming bacteria thus controls the amount of luminal biotin available to the host. We decided to add vitamin B7 (biotin) due to ethical reasons, as both groups of patients should receive a substance that might be beneficial for them and we did not expect vitamin B7 to influence the outcome of the study as no influence on mood symptoms is known to date. This means that individuals receiving probiotic supplements as well as individuals receiving placebo got the same amount of vitamin B7 (biotin) during the study period. Nevertheless, this pathway seems to be upregulated only in individuals receiving probiotic supplement so that we conclude that either some of the bacteria included in the probiotic product increased the availability of biotin in the gut or that this finding is independent of the supplement of biotin and due to the increased production of biotin by bacteria included in the probiotic supplement. As biotin can also be supplied by diet, the question of lifestyle influences also arises in connection to our results. Studies supporting the connection between the quality of diet, gut microbiota and mental health through the regulation of metabolic functions, anti-inflammatory and anti-apoptotic properties and the support of neurogenesis including dietary coaching of patients should, therefore, be an additional independent goal for clinical practice and research in the field of psychiatry [57].

Concerning the missing changes in the levels of zonulin, also in the literature there is only sparse data. To date, there are just two studies on zonulin in depression. For example, the study of Ohlsson et al. [58] suggested higher levels of zonulin only in a subtype of depression (after a recent suicide attempt), while non-suicidal MDD patients showed no differences in zonulin in comparison to healthy controls. The other study by Stevens at al. [59] did not adequately differentiate between patients with depression and anxiety. Due to likely low zonulin levels at baseline, we would not expect a probiotic intervention to show effects on zonulin levels. Our study results confirm the results of other interventional studies that show that multistrain probiotics do not affect zonulin levels [60,61].

Moreover, the upregulation of the Interleukine-17 (IL-17) pathways, shown to have the strongest effect induced by the probiotic intervention, was one of the most important findings of the study. IL-17 is a critical mediator of inflammation and plays a key role in immune activation, and therefore, also plays a role in autorimmune diseases (for reviews see [62,63]). Despite being a modest activator of signaling compared to other inflammatory stimuli, its capacity to synergize with other inflammatory signals makes it a vital inflammatory effector. IL-17 signaling controls inflammation by regulating the expression of inflammatory genes. The IL-17 signature genes are common inflammatory genes regulated by IL-17 and include IL-6, IL-1, granulocyte macrophage colony-stimulating factor (GM-CSF) and tumor-necrose-factor (TNF) [62]. In the context of the microbiome, it is interesting that IL-17-mediated inflammation is critical for microbial clearance [63]. It is known that IL-17 has a dominant protective role in intestinal barrier epithelial integrity by regulating tight junction proteins that connect and stabilize epithelial cell connections [64]. This helps to maintain the barrier and to keep out gut luminal contents and commensal organisms. Furthermore, IL-17 regulates the production of the tight junction protein claudin [65] and IL-17A-dependent regulation of the tight junction protein occludin during epithelial injury is key in limiting excessive permeability and maintaining barrier integrity [64].

## 5. Limitations

Firstly, the relatively strong decrease of depressive symptoms in both groups over the period of 28 days of intake of study medication is a good sign, as the antidepressive inpatient treatment was very effective. Nevertheless, this strong decrease in a relatively small sample might have been one reason why significant differences in psychiatric symptoms between the groups over time have not been detected. In addition, the time of intake of 28 days might be too short to see changes on a clinical level. Secondly, patients were included in the study at the time of admission to hospital. Changes in nutritional habits, due to the hospital meals, might have influenced the results. Thirdly, due to the difference at baseline for smoking status between both groups, it cannot be ruled out that smoking status had a confounding influence on the results.

Furthermore, the intake of biotin, which was added due to ethical reasons, might have influenced different pathways. Due to the high number of females in our study, which was due to the structure of our inpatient setting, the results might reflect more the situation in women than in men. In addition, studies with high samples sizes might help to find changes more easily.

## 6. Conclusions

This study provides further evidence that the intake of probiotic supplements in addition to standard therapy might help to balance microbiota composition in individuals with depressive disorders already early in the treatment period. Furthermore, influences on pathways associated with somatic comorbidities in individuals with depression might positively influence somatic comorbidities and help to better understand associations between somatic and psychiatric diseases in the context of the MGBA.

## Figures and Tables

**Table 1:** Description of the study cohort at baseline (t0).

Description	Intervention Group	Placebo Group	Statistics	
	(*n* = 28)	(*n* = 33)		
	*N (%)*	*N (%)*	*χ* ^2^	*Sig (p)*
Sex (female)	20 (71.4%)	27 (81.8%)	0.925	0.336
Smoking (yes)	9 (32.1%)	19 (57.6%)	3.946	0.047
	Mean (SD)	Mean (SD)	*T*	*Sig (p)*
Age (years)	43.00 (14.31)	40.11 (11.45)	−0.876	0.384
HAMD	15.07 (6.32)	14.73 (4.59)	0.246	0.807
BDI-II	30.75 (8.40)	32.60 (10.93)	−0.719	0.475
BMI (kg/m^2^)	26.29 (5.78)	25.74 (7.29)	−0.319	0.751
Waist-to-hip ratio	0.86 (0.07)	0.84 (0.10)	−0.739	0.463
Education (years)	11.04 (2.87)	10.45 (2.05)	−0.919	0.362
Illness duration (years)	11.40 (13.52)	11.15 (8.34)	−0.090	0.929

Note. HAMD = Hamilton Depression Scale, BDI-II = Beck’s Depression Inventory, BMI = Body Mass Index, SD = Standard Deviation.

**Table 2:** Changes in psychiatric scales.

Scores	Intervention Group (*n* = 28)	Placebo Group (*n* = 30)	Time		Group		Time * Group	
	Mean (SD)	Mean (SD)	*F*	*p*	*F*	*p*	*F*	*p*
HAMD t0	15.07 (6.32)	14.43 (4.41)	47.853	**0.000**	0.482	0.490	0.036	0.850
HAMD t2	9.11 (5.16)	8.13 (6.16)						
BDI-II t0	30.75 (8.40)	32.6 (10.93)	114.635	**0.000**	1.284	0.262	0.196	0.660
BDI-II t2	15.11 (7.91)	18.2 (11.53)						
MSS t0	7.18 (5.67)	8.37 (7.18)	4.882	**0.031**	0.923	.341	0.029	0.866
MSS t2	5.43 (4.61)	6.87 (5.78)						
GSI t0	67.68 (5.68)	67.20 (8.52)	64.293	**0.000**	0.056	0.813	0.882	0.352
GSI t2	58.36 (9.79)	59.83 (10.76)						
PST t0	66.93 (6.72)	66.23 (8.89)	34.866	**0.000**	0.001	0.978	0.476	0.493
PST t2	59.68 (11.28)	60.50 (10.80)						
PSDI t0	65.71 (5.47)	65.20 (8.48)	58.700	**0.000**	0.001	0.977	0.233	0.631
SCL-R PSDI t2	56.07 (9.18)	56.70 (10.68)						
GIQL t0	75.89 (17.07)	76.37 (16.24)	47.841	**0.000**	0.032	0.859	0.018	0.895
GIQL t2	88.81 (17.85)	89.80 (17.22)						

Note. HAMD = Hamilton Depression Scale, BDI-II = Becks Depression Inventory II, MSS = Mania Self Rating Scale, GSI = Global Symptom Index, PST = Positive Symptom Total, PSDI = Positive Symptom Distress Index, GIQL = gastrointestinal quality of life, t0 = time of admission, t2 = after 4 weeks of intervention. Significant differences in bold. * Interaction Time/Group.

**Table 3:** Changes in Zonulin levels.

Zonulin Concentrations	Intervention Group (*n* = 28)	Placebo Group (*n* = 31)	Time		Group		Time * Group	
	Mean (SD)	Mean (SD)	*f*	*p*	*f*	*p*	*f*	*p*
Zonulin [ng/mL] t0	46.801 (15.957)	52.007 (10.906)	0.560	0.457	1.662	0.202	0.426	0.516
Zonulin [ng/mL] t2	50.161 (15.909)	52.236 (13.827)						

Note. Time * Group = Interaction between group and time.

**Table 4:** KEGG-pathways.

KEGG Pathway	Probiotics Versus Placebo, T2, Effect Size	Name of Pathway
**KO04657**	0.463	IL-17 signaling pathway
**KO00780**	0.432	Biotin (Vitamin B7) metabolism
**KO04910**	0.424	Insulin signaling pathway
**KO00750**	0.410	Vitamin B6 metabolism
**KO00500**	0.404	Starch and sucrose metabolism
**KO05010**	0.384	Alzheimer disease
**KO00730**	0.370	Thiamine (vitamin B1) metabolism
**KO00400**	0.368	Phenylalanine, tyrosine and tryptophan biosynthesis
**KO00190**	0.363	Oxidative phosphorylation
**KO00770**	0.362	Pantothenate and CoA biosynthesis
**KO01100**	0.362	Metabolic pathways
**KO00760**	0.359	Nicotinate and nicotinamide metabolism
**KO00471**	0.358	D-Glutamine and D-glutamate metabolism
**KO01040**	0.355	Biosynthesis of unsaturated fatty acids
**KO00620**	0.349	Pyruvate metabolism
**KO00290**	0.340	Valine, leucine and isoleucine biosynthesis
**KO00061**	0.332	Fatty acid biosynthesis
**KO00010**	0.326	Glycolysis/Gluconeogenesis
**KO04724**	0.326	Glutamatergic synapse
**KO01212**	0.323	Fatty acid metabolism
**KO00640**	0.319	Propanoate metabolism
**KO00020**	0.309	Citrate cycle (TCA cycle)
**KO04727**	0.294	GABAergic synapse

Note. IL = interleukin, GABA = gamma-Aminobutyric acid.
